# Editorial for Special Issue “Plasmon Assisted Near-Field Manipulation and Photocatalysis”

**DOI:** 10.3390/nano13081427

**Published:** 2023-04-21

**Authors:** Zhenglong Zhang

**Affiliations:** School of Physics and Information Technology, Shaanxi Normal University, Xi’an 710062, China; zlzhang@snnu.edu.cn

Accurately establishing the near field is crucial to enhancing optical manipulation and resolution, and is pivotal to the application of nanoparticles in the field of photocatalysis. A novel type of modulated optical field that enables the multi-dimensional control of the amplitude, polarization and phase can be obtained via the precise manipulation of the plasmonic near field of metal nanostructures. Meanwhile, the energy stored in the plasmonic field can induce hot carriers in the metal, which ultimately dissipate by coupling to the phonon modes of the metal nanoparticles, resulting in an elevated lattice temperature.

The plasmonic nearfield, hot carriers and their heating effects can catalyze the chemical reactions of reactants, including molecules and nanomaterials. Firstly, the chemical efficiency can be enhanced by the plasmonic near field due to its elevated photon density. Secondly, the hot carriers induced by plasmon decay can transfer to the reactant via the indirect electron transfer or direct electron excitation process, and the specific chemical reaction channels can be selectively enhanced by controlling the energy distribution of hot carriers. Thirdly, the local thermal effect that is followed by plasmon decay offers opportunities to facilitate the chemical reactions of molecules and induce the crystal growth and transformation of nanomaterials at room temperature. As a new class of photocatalysts, plasmonic noble metal nanoparticles that possess the unique ability to harvest light energy across the entire visible spectrum and produce effective energy conversion have been explored as a promising novel direction in the amelioration of the energy crisis. These opportunities have motivated this Special Issue, entitled ‘Plasmon-Assisted Near-Field Manipulation and Photocatalysis’, which has attracted research and review papers related to a variety of emerging nanomaterials, as outlined below.

Noble metal (Au/Ag/Cu/Al) nanostructures can produce surface plasmon resonance to promote or facilitate chemical reactions, as well as photocatalytic materials. In particular, Ag/Au nanoislands (NIs) and Ag NIs/Au film composite systems were designed, and their thermo-catalysis performance was investigated using the luminescence of Eu^3+^ as a probe [1]. It was discovered that the metal NIs can also generate strong localized heat in low-temperature environments, enabling the transition of NaYF_4_ to Y_2_O_3_. Furthermore, anti-oxidation was realized by depositing gold on the surface of silver, resulting in the relative stability of the constructed complex. These investigations can provide an enhanced understanding of the surface plasmon catalysis process and extend the potential applications of metal NIs. For utilization in an alternative application, self-assembled Al NIs with a thin alumina layer were designed with a plasmonic photothermal structure in order to achieve nanocrystal transformation via multi-wavelength excitation [2]. The Al NIs with an alumina layer demonstrated excellent photothermal conversion efficiency even in low-temperature environments, and their efficacy did decline significantly after storage in air for three months. Such an inexpensive Al/Al_2_O_3_ structure with a multi-wavelength response provides an efficient platform through which to achieve rapid nanocrystal transformation and fulfil its potential application in the wide-band absorption of solar energy.

Oxide-supported noble metal nanoparticles, which are one of the primary photocatalytic nanomaterials, have been investigated for their potential ability to enhance the stability and diminish the cost of photocatalysts. In particular, an Ag-nanoparticles-doped porous ZnO photocatalyst was prepared [3]. Under visible light irradiation, the heterostructure showed excellent catalytic activity over 4-nitrophenol due to the hot electrons induced by the localized surface plasmon resonance of Ag nanoparticles; this provides a novel heterostructure photocatalyst with the potential to be applied in solar energy and pollutant disposal. In addition, the optical properties of substitutional-doped aluminum nitride (AlN) were studied via multi-scale computational simulation methods, combined with density functional theory and finite element analysis [4]. It was discovered that a strong AlN surface plasmon resonance could be obtained in the near-infrared region by applying various alkali metal doping configurations, which not only improve the application of multi-scale computational simulations in quantum surface plasmons, but also promote the application of AlN in the field of surface-enhanced linear and non-linear optical spectroscopy.

Due to the large inherent loss of metals that occurs in phase matching, and its further limitations, a larger Q-factor cannot be obtained by utilizing traditional optical cavity modes and devices based on surface plasmon resonance. A silicon square-hole nano-disk array device was proposed in order to realize multi-Fano resonances with a high Q-factor, narrow line width, large modulation depth and enhanced near-field enhancement, which could provide the basis for the application of a novel method in the realms of multi-wavelength communications, lasing, and nonlinear optical devices [5]. Then, a nested composite structured multifunctional metasurface zone plate was designed and fabricated by integrating the metasurface onto the surface of the multi-level diffraction lens rings [6]. Based on the global optimization mathematical iterative method, the height distribution of the multifunctional metasurface zone plate was optimized in order to realize an extremely efficient achromatic broadband focus. This combination enhances the degree of freedom that exists in micro–nano optical design, and is expected to be applied in multifunctional focusing devices, polarization imaging, and various other fields. Lastly, the self-organizing process of component and array manufacturing was combined with imprinting technology in order to construct a cheap and reproducible flexible polyvinyl alcohol nanocavity array that is decorated with the silver nanoparticles [7]. The substrate exhibited excellent mechanical stability in bending experiments, and was able to achieve low-cost, high-sensitivity, uniform and favorable surface-enhanced Raman scattering detection, particularly in regard to situ detection; furthermore, it demonstrated promise in food safety and biomedicine applications.

As a plasmonic photocatalytic mechanism, the photothermal properties of nanomaterials have received widespread attention due to their broad applicative potential. The near-field and photo-thermal temperature distribution of a nanoparticle array was numerically investigated by considering the scattering light field among particles [8]. It has been determined that the position of the ‘hot spots’ does not rotate with the polarization direction of the incident light and always remains in the particle gaps along the line between the particle centers, which provides theoretical considerations for the near-field manipulation and photo-thermal applications of nanoarrays.

Finally, it was determined that the surface plasmon could strongly confine electromagnetic fields near the metal nanostructures in order to generate a localized near field, which has been widely utilized in surface-enhanced spectroscopy and nonlinear optics. An overview of the mechanism of surface plasmon and of near-field nonlinear effects is offered in one paper published in this volume, and describes some of the latest research that focuses on the applications of nonlinear optical microscopy systems [9]. Furthermore, the enhanced near field, hot carriers and localized thermal effect play an important role in photocatalysis. The other review paper is focused upon surface-plasmon-assisted photocatalysis, including nanomaterial reshaping, growth and transformation [10]. The current status of and perspectives on the future of plasmonic photocatalysis are reviewed, which will promote the development of surface plasmon in regard to the regulation of nanomaterials.

Overall, this volume provides a selected collection of papers that covers various aspects of plasmonic near-field manipulation and photocatalysis; we sincerely hope that the reader will benefit from such an informative and insightful Special Issue. 
